# *N*-(2-Formyl­phen­yl)-4-toluene­sulfonamide: a second monoclinic polymorph

**DOI:** 10.1107/S1600536812009403

**Published:** 2012-03-10

**Authors:** S. Murugavel, N. Manikandan, D. Kannan, M. Bakthadoss

**Affiliations:** a Department of Physics, Thanthai Periyar Government Institute of Technology, Vellore 632 002, India; b Department of Physics, Bharathidasan Engineering College, Nattrampalli, Vellore 635 854, India; c Department of Organic Chemistry, University of Madras, Maraimalai Campus, Chennai 600 025, India

## Abstract

The title compound, C_14_H_13_NO_3_S, (I), is a second monoclinic polymorph. The original polymorph, (II), was reported by Mahía *et al.* [*Acta Cryst.* (1999), C**55**, 2158–2160]. Polymorph (II) crystalllized in the space group *P*2_1_/*c* (*Z* = 4), whereas the title polymorph (I) occurs in the space group *P*2_1_/*n* (*Z* = 4). The dihedral angle between the two aromatic rings is 75.9 (1)° in (I) compared to 81.9 (1)° for (II). In both polymorphs, two *S*(6) rings are generated by intra­molecular N—H⋯O and C—H⋯O hydrogen bonds, resulting in similar mol­ecular geometries. However, the two polymorphs differ concerning their crystal packing. In (I), mol­ecules are linked into *C*(8) zigzag chains along the *b* axis by C—H⋯O hydrogen bonds, whereas in (II) mol­ecules are linked by C—H⋯O hydrogen bonds, forming *C*(7) chains along the *b* axis. The title polymorph is further stabilized by inter­molecular C—H⋯π and π–π inter­actions [centroid–centroid distance = 3.814 (1) Å]. These inter­actions are not evident in polymorph (II).

## Related literature

For biological applications of sulfonamides, see: Connor (1998 ▶); Berredjem *et al.* (2000 ▶); Lee & Lee (2002 ▶); Xiao & Timberlake (2000 ▶). For the first monoclinic polymorph, see: Mahía *et al.* (1999 ▶). For a related structure, see: Zhang *et al.* (2010 ▶). For hydrogen-bond motifs, see: Bernstein *et al.* (1995 ▶).
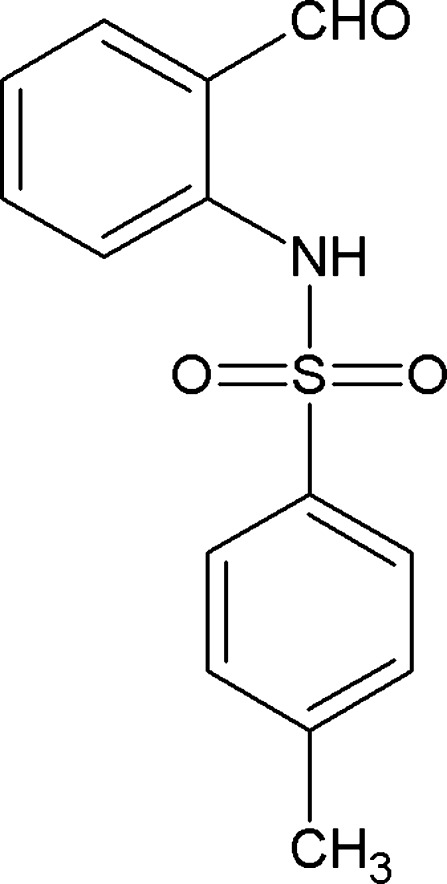


## Experimental

### 

#### Crystal data

C_14_H_13_NO_3_S
*M* *_r_* = 275.31
Monoclinic, 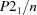

*a* = 11.5409 (4) Å
*b* = 8.1345 (2) Å
*c* = 14.1115 (5) Å
β = 97.294 (2)°
*V* = 1314.06 (7) Å^3^
*Z* = 4
Mo *K*α radiation
μ = 0.25 mm^−1^
*T* = 293 K
0.25 × 0.23 × 0.18 mm

#### Data collection

Bruker APEXII CCD diffractometer
Absorption correction: multi-scan (*SADABS*; Sheldrick, 1996 ▶) *T*_min_ = 0.940, *T*_max_ = 0.956
16047 measured reflections
4254 independent reflections
2678 reflections with *I* > 2σ(*I*)
*R*_int_ = 0.029

#### Refinement

*R*[*F* ^2^ > 2σ(*F* ^2^)] = 0.049
*wR*(*F* ^2^) = 0.155
*S* = 1.02
4254 reflections
174 parameters
H-atom parameters constrained
Δρ_max_ = 0.30 e Å^−3^
Δρ_min_ = −0.40 e Å^−3^

### 

Data collection: *APEX2* (Bruker, 2004 ▶); cell refinement: *APEX2* and *SAINT* (Bruker, 2004 ▶); data reduction: *SAINT* and *XPREP* (Bruker, 2004 ▶); program(s) used to solve structure: *SHELXS97* (Sheldrick, 2008 ▶); program(s) used to refine structure: *SHELXL97* (Sheldrick, 2008 ▶); molecular graphics: *ORTEP-3* (Farrugia, 1997 ▶); software used to prepare material for publication: *SHELXL97* and *PLATON* (Spek, 2009 ▶).

## Supplementary Material

Crystal structure: contains datablock(s) global, I. DOI: 10.1107/S1600536812009403/im2360sup1.cif

Structure factors: contains datablock(s) I. DOI: 10.1107/S1600536812009403/im2360Isup2.hkl

Supplementary material file. DOI: 10.1107/S1600536812009403/im2360Isup3.cml

Additional supplementary materials:  crystallographic information; 3D view; checkCIF report

## Figures and Tables

**Table 1:** Hydrogen-bond geometry (Å, °) *Cg*1 is the centroid of the C8–C13 benzene ring.

*D*—H⋯*A*	*D*—H	H⋯*A*	*D*⋯*A*	*D*—H⋯*A*
N1—H1⋯O1	0.86	1.94	2.655 (2)	140
C2—H2⋯O2	0.93	2.48	3.059 (2)	120
C14—H14*C*⋯O3^i^	0.96	2.52	3.439 (3)	161
C5—H5⋯*Cg*1^ii^	0.93	2.82	3.658 (2)	150

Symmetry codes: (i) 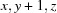; (ii) 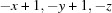.

**Fig. 1.**
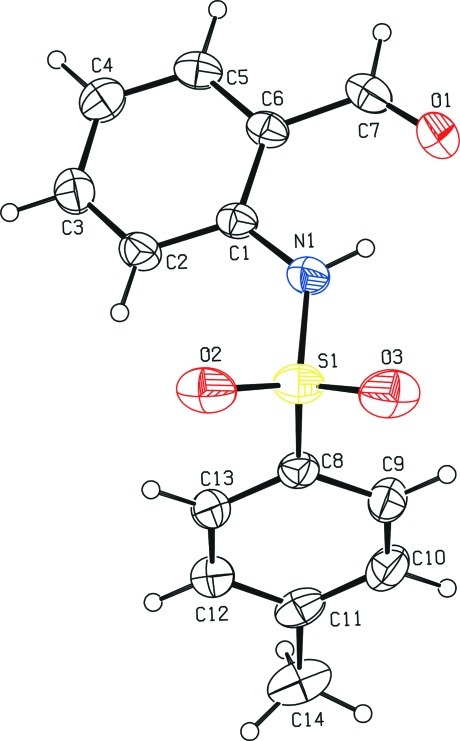
Molecular structure of the title compound with displacement ellipsoids drawn at the 30% probability level. H atoms are presented as a small spheres of arbitrary radius.

**Fig. 2.**
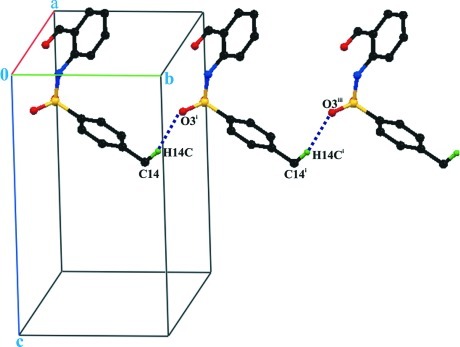
Part of the crystal structure of (I) showing C—H···O hydrogen bonds (blue dotted lines), with the formation of C(8) chains along the b axis. [Symmetry codes: (i) x, 1 + y, z; (iii) x, 2 + y, z].

**Fig. 3.**
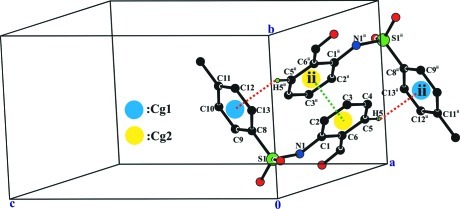
A view of the C—H···π and π—π interactions (dotted lines) in the crystal structure of the title compound. Cg1 and Cg2 denotes centroids of the C8–C13 benzene ring and C1–C6 benzene ring, respectively. [Symmetry code: (ii) 1 - x, 1 - y, -z].

## supplementary crystallographic information

### Comment

Sulfonamides are an important category of pharmaceutical compounds with a broad spectrum of biological activities such as herbicidal, anti-malarial, anti-convulsant and anti-hypertensive (Connor, 1998; Xiao & Timberlake, 2000; Berredjem *et al.*, 2000; Lee & Lee, 2002). In this work we report the crystal structure of the title compound (Fig 1, Alternative name: 2–tosylaminobenzaldehyde), which is the second monoclinic polymorph reported.

The original polymorph (compound (II)) was previously reported by Mahía *et al.* (1999) and was shown to crystallize in the monoclinic space group *P*2_1_/*c*, with a = 13.899 (7), b = 8.237 (4), c = 12.063 (6) Å, β = 105.899 (1)° and *Z* = 4. In the present work, the title compound crystallized in the space group *P*2_1_/*n* with a = 11.5409 (4), b = 8.1345 (2), c = 14.1115 (5) Å, β = 97.294 (2)° and *Z* = 4. The dihedral angle between the two aromatic rings is 75.9 (1)° in (I) compared to 81.9 (1)° for (II). The bond distances in the molecule are normal and comparable to those in the previously published polymorph and in a closely related sulfonamide derivative (Zhang *et al.*, 2010).

In both polymorphs, the molecular packing is stabilized by intramolecular N1—H1···O1 and C2—H2···O2 hydrogen bonds, generating two S(6) rings (Bernstein *et al.*, 1995) (Table 1). However, the two polymorphs differ concerning their crystal packing. In compound (II) molecules are linked to form C(7) chains along the *b* axis by intermolecular C3—H3···O3 hydrogen bonds whereas in the title compound (I), molecules are linked by intermolecular C14—H14C···O3 hydrogen bonds to form C(8) zigzag chains along the *b* axis (Fig. 2). The crystal packing (Fig. 3) is further stabilized by C—H···π interactions between a formylphenyl H atom and the benzene ring (C8–C13) of a neighbouring molecule, with a C5—H5···*Cg*1^ii^ distance of 3.658 (2) Å (Table 1; *Cg*1 is the centroid of the C8–C13 benzene ring, Symmetry code: ii = *1 - x, 1 - y, -z*). Additional stability arises from aromatic π—π interaction between the benzene rings of neighbouring molecules, with *Cg*2—*Cg*2^ii^ distance of 3.814 (1) Å (Fig. 3; *Cg*2 is the centroid of the C1—C6 benzene ring, Symmetry code: ii = *1 - x, 1 - y, -z*). The C—H···π and π—π interactions are not evident in compound (II).

### Experimental

To a stirred solution of (2-aminophenyl)methanol in chloroform, 1.5 equiv of pyridine and 1.5 equiv of 4-methylbenzene-1-sulfonyl chloride were added at room temperature over a period of 18 h. The resulting brown colored solution was quenched with aqueous HCl. After workup a pale brownish solid was obtained. The combined organic fractions were dried over Na_2_SO_4_ and partially concentrated under reduced pressure at 25°C. The product was precipitated with diethylether. Further, MnO_2_ was added to a solution of 2-tosylaminobenzyl alcohol in dry 1,2-dichloroethane solvent under nitrogen atmosphere. The suspension was stirred at 80°C in reflux condition for 5 h and filtered through Celite. The filtrate was partially concentrated on a rotatory evaporator at 25°C and the product was precipitated with diethylether. Recystallization of the product from CH_2_Cl_2_ yielded pale yellow crystals of the title compound (Yield: 86%). Crystals of the original polymorph were prepared by evaporation of CHCl_3_ from a solution of the title compound at room temperature (Mahía *et al.*, 1999).

### Refinement

H atoms were positioned geometrically (N–H = 0.86 Å and C–H = 0.93–0.96 Å and allowed to ride on their parent atoms, with *U*_iso_(H) =1.5*U*_eq_ for methyl H atoms and 1.2*U*_eq_(C) for other H atoms.

### Crystal data

**Table:**

C_14_H_13_NO_3_S	*F*(000) = 576
*M**_r_* = 275.31	*D*_x_ = 1.392 Mg m^−^^3^
Monoclinic, *P*2_1_/*n*	Mo *K*α radiation, λ = 0.71073 Å
Hall symbol: -P 2yn	Cell parameters from 4561 reflections
*a* = 11.5409 (4) Å	θ = 2.2–32.0°
*b* = 8.1345 (2) Å	µ = 0.25 mm^−^^1^
*c* = 14.1115 (5) Å	*T* = 293 K
β = 97.294 (2)°	Block, pale yellow
*V* = 1314.06 (7) Å^3^	0.25 × 0.23 × 0.18 mm
*Z* = 4	

### Data collection

**Table:**

Bruker APEXII CCD diffractometer	4254 independent reflections
Radiation source: fine-focus sealed tube	2678 reflections with *I* > 2σ(*I*)
Graphite monochromator	*R*_int_ = 0.029
Detector resolution: 10.0 pixels mm^-1^	θ_max_ = 32.0°, θ_min_ = 2.2°
ω scans	*h* = −15→16
Absorption correction: multi-scan (*SADABS*; Sheldrick, 1996)	*k* = −11→12
*T*_min_ = 0.940, *T*_max_ = 0.956	*l* = −16→21
16047 measured reflections	

### Refinement

**Table:**

Refinement on *F*^2^	Secondary atom site location: difference Fourier map
Least-squares matrix: full	Hydrogen site location: inferred from neighbouring sites
*R*[*F*^2^ > 2σ(*F*^2^)] = 0.049	H-atom parameters constrained
*wR*(*F*^2^) = 0.155	*w* = 1/[σ^2^(*F*_o_^2^) + (0.0735*P*)^2^ + 0.2545*P*] where *P* = (*F*_o_^2^ + 2*F*_c_^2^)/3
*S* = 1.02	(Δ/σ)_max_ < 0.001
4254 reflections	Δρ_max_ = 0.30 e Å^−^^3^
174 parameters	Δρ_min_ = −0.40 e Å^−^^3^
0 restraints	Extinction correction: *SHELXL97* (Sheldrick, 2008), Fc^*^=kFc[1+0.001xFc^2^λ^3^/sin(2θ)]^-1/4^
Primary atom site location: structure-invariant direct methods	Extinction coefficient: 0.023 (3)

### Special details

**Table:**

Geometry. All e.s.d.'s (except the e.s.d. in the dihedral angle between two l.s. planes) are estimated using the full covariance matrix. The cell e.s.d.'s are taken into account individually in the estimation of e.s.d.'s in distances, angles and torsion angles; correlations between e.s.d.'s in cell parameters are only used when they are defined by crystal symmetry. An approximate (isotropic) treatment of cell e.s.d.'s is used for estimating e.s.d.'s involving l.s. planes.
Refinement. Refinement of *F*^2^ against ALL reflections. The weighted *R*-factor *wR* and goodness of fit *S* are based on *F*^2^, conventional *R*-factors *R* are based on *F*, with *F* set to zero for negative *F*^2^. The threshold expression of *F*^2^ > σ(*F*^2^) is used only for calculating *R*-factors(gt) *etc*. and is not relevant to the choice of reflections for refinement. *R*-factors based on *F*^2^ are statistically about twice as large as those based on *F*, and *R*- factors based on ALL data will be even larger.

### Fractional atomic coordinates and isotropic or equivalent isotropic displacement parameters (Å^2^)

**Table:**

	*x*	*y*	*z*	*U*_iso_*/*U*_eq_	
S1	0.52951 (5)	0.12891 (6)	0.23059 (3)	0.06087 (19)	
C1	0.56496 (14)	0.25944 (19)	0.05828 (10)	0.0454 (4)	
C6	0.51658 (14)	0.27675 (19)	−0.03793 (11)	0.0472 (4)	
N1	0.50096 (14)	0.1677 (2)	0.11710 (10)	0.0566 (4)	
H1	0.4372	0.1256	0.0892	0.068*	
C5	0.57925 (17)	0.3623 (2)	−0.10019 (12)	0.0580 (4)	
H5	0.5484	0.3721	−0.1641	0.070*	
O1	0.33743 (12)	0.13376 (18)	−0.03182 (11)	0.0708 (4)	
C8	0.49992 (15)	0.3098 (2)	0.29018 (11)	0.0497 (4)	
C13	0.58899 (15)	0.4156 (2)	0.32385 (12)	0.0563 (4)	
H13	0.6657	0.3916	0.3151	0.068*	
C7	0.40286 (17)	0.2107 (2)	−0.07598 (13)	0.0608 (5)	
H7	0.3780	0.2293	−0.1404	0.073*	
C2	0.67100 (15)	0.3343 (2)	0.08926 (12)	0.0556 (4)	
H2	0.7026	0.3273	0.1531	0.067*	
O2	0.65042 (14)	0.0934 (2)	0.25269 (11)	0.0804 (5)	
C11	0.45109 (18)	0.5950 (2)	0.38535 (13)	0.0614 (5)	
C12	0.56359 (17)	0.5566 (3)	0.37043 (14)	0.0627 (5)	
H12	0.6239	0.6281	0.3925	0.075*	
C4	0.68487 (18)	0.4321 (3)	−0.06949 (15)	0.0640 (5)	
H4	0.7262	0.4877	−0.1120	0.077*	
C10	0.36292 (18)	0.4862 (3)	0.35138 (15)	0.0680 (5)	
H10	0.2864	0.5098	0.3608	0.082*	
O3	0.44396 (17)	0.0087 (2)	0.24676 (12)	0.0891 (5)	
C3	0.72931 (17)	0.4190 (2)	0.02550 (15)	0.0612 (5)	
H3	0.8004	0.4686	0.0470	0.073*	
C9	0.38592 (17)	0.3451 (3)	0.30435 (14)	0.0626 (5)	
H9	0.3257	0.2736	0.2821	0.075*	
C14	0.4263 (3)	0.7500 (3)	0.43715 (17)	0.0867 (7)	
H14A	0.4874	0.7685	0.4888	0.130*	
H14B	0.3530	0.7394	0.4620	0.130*	
H14C	0.4226	0.8412	0.3937	0.130*	

### Atomic displacement parameters (Å^2^)

**Table:**

	*U*^11^	*U*^22^	*U*^33^	*U*^12^	*U*^13^	*U*^23^
S1	0.0805 (4)	0.0561 (3)	0.0451 (2)	0.0081 (2)	0.0047 (2)	0.00904 (18)
C1	0.0500 (9)	0.0445 (8)	0.0410 (7)	0.0082 (7)	0.0029 (6)	−0.0031 (6)
C6	0.0532 (9)	0.0437 (8)	0.0427 (8)	0.0071 (7)	−0.0011 (6)	−0.0044 (6)
N1	0.0595 (9)	0.0669 (9)	0.0423 (7)	−0.0054 (7)	0.0017 (6)	0.0015 (6)
C5	0.0707 (12)	0.0595 (10)	0.0421 (8)	0.0054 (8)	0.0008 (8)	0.0025 (7)
O1	0.0607 (8)	0.0794 (10)	0.0696 (9)	−0.0118 (7)	−0.0021 (7)	−0.0111 (7)
C8	0.0514 (9)	0.0599 (9)	0.0378 (7)	0.0042 (7)	0.0054 (6)	0.0084 (7)
C13	0.0441 (9)	0.0767 (12)	0.0476 (9)	0.0044 (8)	0.0039 (7)	0.0005 (8)
C7	0.0642 (12)	0.0651 (11)	0.0489 (9)	0.0029 (9)	−0.0086 (8)	−0.0085 (8)
C2	0.0519 (10)	0.0678 (11)	0.0445 (8)	0.0046 (8)	−0.0039 (7)	−0.0020 (7)
O2	0.0930 (11)	0.0843 (10)	0.0605 (8)	0.0407 (8)	−0.0041 (7)	0.0065 (7)
C11	0.0777 (13)	0.0623 (11)	0.0476 (9)	0.0109 (9)	0.0218 (9)	0.0132 (8)
C12	0.0633 (11)	0.0707 (12)	0.0548 (10)	−0.0064 (9)	0.0098 (8)	−0.0037 (9)
C4	0.0695 (12)	0.0635 (11)	0.0608 (11)	−0.0013 (9)	0.0150 (9)	0.0072 (9)
C10	0.0535 (11)	0.0891 (15)	0.0649 (12)	0.0125 (10)	0.0210 (9)	0.0108 (10)
O3	0.1354 (14)	0.0640 (9)	0.0700 (10)	−0.0193 (9)	0.0206 (9)	0.0124 (7)
C3	0.0514 (10)	0.0642 (11)	0.0672 (12)	−0.0025 (8)	0.0043 (8)	−0.0027 (9)
C9	0.0498 (10)	0.0785 (13)	0.0606 (11)	−0.0063 (9)	0.0115 (8)	0.0067 (9)
C14	0.128 (2)	0.0684 (13)	0.0712 (14)	0.0182 (14)	0.0426 (14)	0.0085 (11)

### Geometric parameters (Å, º)

**Table:**

S1—O2	1.4204 (16)	C7—H7	0.9300
S1—O3	1.4285 (16)	C2—C3	1.375 (3)
S1—N1	1.6245 (14)	C2—H2	0.9300
S1—C8	1.7500 (18)	C11—C12	1.377 (3)
C1—C2	1.387 (2)	C11—C10	1.387 (3)
C1—N1	1.395 (2)	C11—C14	1.503 (3)
C1—C6	1.408 (2)	C12—H12	0.9300
C6—C5	1.393 (2)	C4—C3	1.377 (3)
C6—C7	1.456 (3)	C4—H4	0.9300
N1—H1	0.8600	C10—C9	1.369 (3)
C5—C4	1.364 (3)	C10—H10	0.9300
C5—H5	0.9300	C3—H3	0.9300
O1—C7	1.212 (2)	C9—H9	0.9300
C8—C13	1.378 (3)	C14—H14A	0.9600
C8—C9	1.386 (3)	C14—H14B	0.9600
C13—C12	1.372 (3)	C14—H14C	0.9600
C13—H13	0.9300		
			
O2—S1—O3	120.36 (11)	C3—C2—C1	119.95 (16)
O2—S1—N1	109.05 (9)	C3—C2—H2	120.0
O3—S1—N1	103.45 (9)	C1—C2—H2	120.0
O2—S1—C8	108.16 (9)	C12—C11—C10	117.85 (19)
O3—S1—C8	108.55 (9)	C12—C11—C14	120.4 (2)
N1—S1—C8	106.44 (8)	C10—C11—C14	121.76 (19)
C2—C1—N1	124.04 (14)	C13—C12—C11	121.71 (19)
C2—C1—C6	119.08 (15)	C13—C12—H12	119.1
N1—C1—C6	116.87 (15)	C11—C12—H12	119.1
C5—C6—C1	118.94 (15)	C5—C4—C3	118.96 (18)
C5—C6—C7	117.88 (15)	C5—C4—H4	120.5
C1—C6—C7	123.17 (16)	C3—C4—H4	120.5
C1—N1—S1	129.33 (12)	C9—C10—C11	121.57 (18)
C1—N1—H1	115.3	C9—C10—H10	119.2
S1—N1—H1	115.3	C11—C10—H10	119.2
C4—C5—C6	121.47 (16)	C2—C3—C4	121.53 (18)
C4—C5—H5	119.3	C2—C3—H3	119.2
C6—C5—H5	119.3	C4—C3—H3	119.2
C13—C8—C9	120.11 (18)	C10—C9—C8	119.3 (2)
C13—C8—S1	120.54 (13)	C10—C9—H9	120.3
C9—C8—S1	119.35 (15)	C8—C9—H9	120.3
C12—C13—C8	119.45 (17)	C11—C14—H14A	109.5
C12—C13—H13	120.3	C11—C14—H14B	109.5
C8—C13—H13	120.3	H14A—C14—H14B	109.5
O1—C7—C6	126.30 (17)	C11—C14—H14C	109.5
O1—C7—H7	116.9	H14A—C14—H14C	109.5
C6—C7—H7	116.9	H14B—C14—H14C	109.5
			
C2—C1—C6—C5	2.9 (2)	C9—C8—C13—C12	−0.8 (3)
N1—C1—C6—C5	−177.73 (15)	S1—C8—C13—C12	−179.94 (14)
C2—C1—C6—C7	−176.46 (16)	C5—C6—C7—O1	−179.95 (18)
N1—C1—C6—C7	2.9 (2)	C1—C6—C7—O1	−0.6 (3)
C2—C1—N1—S1	1.3 (3)	N1—C1—C2—C3	178.36 (16)
C6—C1—N1—S1	−178.02 (13)	C6—C1—C2—C3	−2.3 (3)
O2—S1—N1—C1	−41.57 (18)	C8—C13—C12—C11	0.6 (3)
O3—S1—N1—C1	−170.79 (16)	C10—C11—C12—C13	−0.2 (3)
C8—S1—N1—C1	74.91 (17)	C14—C11—C12—C13	179.64 (18)
C1—C6—C5—C4	−1.3 (3)	C6—C5—C4—C3	−0.9 (3)
C7—C6—C5—C4	178.06 (17)	C12—C11—C10—C9	−0.1 (3)
O2—S1—C8—C13	18.43 (17)	C14—C11—C10—C9	−179.92 (19)
O3—S1—C8—C13	150.58 (15)	C1—C2—C3—C4	0.1 (3)
N1—S1—C8—C13	−98.64 (15)	C5—C4—C3—C2	1.5 (3)
O2—S1—C8—C9	−160.77 (14)	C11—C10—C9—C8	−0.1 (3)
O3—S1—C8—C9	−28.61 (16)	C13—C8—C9—C10	0.5 (3)
N1—S1—C8—C9	82.16 (15)	S1—C8—C9—C10	179.68 (15)

### Hydrogen-bond geometry (Å, º)

Cg1 is the centroid of the C8–C13 benzene ring.

**Table:**

*D*—H···*A*	*D*—H	H···*A*	*D*···*A*	*D*—H···*A*
N1—H1···O1	0.86	1.94	2.655 (2)	140
C2—H2···O2	0.93	2.48	3.059 (2)	120
C14—H14*C*···O3^i^	0.96	2.52	3.439 (3)	161
C5—H5···*Cg*1^ii^	0.93	2.82	3.658 (2)	150

Symmetry codes: (i) *x*, *y*+1, *z*; (ii) −*x*+1, −*y*+1, −*z*.
